# The Population Structure and Diversity of Eggplant from Asia and the Mediterranean Basin

**DOI:** 10.1371/journal.pone.0073702

**Published:** 2013-09-06

**Authors:** Fabio Cericola, Ezio Portis, Laura Toppino, Lorenzo Barchi, Nazareno Acciarri, Tommaso Ciriaci, Tea Sala, Giuseppe Leonardo Rotino, Sergio Lanteri

**Affiliations:** 1 DISAFA - Plant Genetics and Breeding, University of Torino, Grugliasco, Torino, Italy; 2 CRA-ORL - Research Unit for Vegetable Crops, Montanaso Lombardo, Lodi, Italy; 3 CRA-ORA - Research Unit for Vegetable Crops, Monsampolo del Tronto, Ascoli Piceno, Italy; Nanjing Agricultural University, China

## Abstract

A collection of 238 eggplant breeding lines, heritage varieties and selections within local landraces provenanced from Asia and the Mediterranean Basin was phenotyped with respect to key plant and fruit traits, and genotyped using 24 microsatellite loci distributed uniformly throughout the genome. STRUCTURE analysis based on the genotypic data identified two major sub-groups, which to a large extent mirrored the provenance of the entries. With the goal to identify true-breeding types, 38 of the entries were discarded on the basis of microsatellite-based residual heterozygosity, along with a further nine which were not phenotypically uniform. The remaining 191 entries were scored for a set of 19 fruit and plant traits in a replicated experimental field trial. The phenotypic data were subjected to principal component and hierarchical principal component analyses, allowing three major morphological groups to be identified. All three morphological groups were represented in both the “Occidental” and the “Oriental” germplasm, so the correlation between the phenotypic and the genotypic data sets was quite weak. The relevance of these results for evolutionary studies and the further improvement of eggplant are discussed. The population structure of the core set of germplasm shows that it can be used as a basis for an association mapping approach.

## Introduction

Eggplant (*Solanum melongena* L.) belongs to the large Solanaceae family, which also includes a number of other significant crop species, in particular tomato, potato, sweet and hot peppers and tobacco. Unlike all of the latter, eggplant is an Old World species. Lester et al. [1] have suggested that the eggplant’s pre-domestication ancestor was the subtropical species *S. incanum*, a native of north Africa and West Asia which is being used in eggplant breeding programs as a source of variation for phenolics content and resistance to drought [2]; others have postulated that the ancestor was rather *S. undatum* [3], [4]. However, recent morphological and molecular work has shown that species-level differences exist between *S. incanum* and *S. melongena* while, on the basis of a new nomenclature, *S. undatum* and *S.cumingii* have been reclassified as *S. insanum*. The latter, distributed from India to SE Asia, and found also in Madagascar and Mauritius, is fully inter-fertile with *S. melongena* and is considered almost certainly its wild progenitor [2]. Sanskrit documents have revealed that the domestication of eggplant was achieved around 100–300 BCE and archaeological records, based on the analysis of microfossils starch grains, suggest that eggplant was present in the diet of inhabitants of the Indus valley during Harappan civilisation, thus Rajasthan may have been an area of domestication [5]. On the other hand the use of eggplant as a vegetable crop was described in Chinese literature dating to 59 BCE [6]. The crop spread westwards to Persia, was unknown by the ancient Greeks and Romans, and was introduced to the Mediterranean Basin by Muslim invaders in the 7th to 8th century CE [7].

The global production of eggplant is estimated to be around 46 Mt (http://faostat.fao.org); it represents an economically and nutritionally important crop in Asia and southern Europe. The bulk of production is concentrated in China, India, Iran, Egypt and Turkey, with Italy representing the most important European producer (http://faostat.fao.org). Eggplant is highly regarded as a source of antioxidants [8], in particular flavonoids and the phenolic chlorogenic acid [9], [10]. These compounds are present in both the fruit’s flesh and skin [11] and their content and profile are developmentally regulated during fruit ripening [12]. Fruit extracts have been shown to have anti-oxidant [13], hepato-protective [14], anti-carcinoma [15], anti-microbial, anti-LDL. anti-viral [16]–[18] and cardio-protective properties [19].

Selection and breeding over some hundreds of years has resulted in the elaboration of a large number of eggplant varieties. These are conventionally grouped as “Occidental” (preferred – grown in North Africa, Europe and the Americas) and “Oriental” eggplants (East and Southeast Asia). They vary from one another both with respect to their overall plant morphology and physiology, with their fruit size, color and shape being particularly distinctive. Fruit color can be cream, green, red, reddish-purple, dark purple or black, and some varieties produce fruit which is striped. Global trade is concentrated on an diminishing number of elite varieties [20]. These include F_1_ hybrids [7] which through their expression of heterosis for yield and their unique genetic status, have become extremely attractive for seed suppliers and breeders [21], [22]. As a result of the growing dominance of commercial hybrids, the genetic diversity of material in cultivation finds itself under some pressure; the conservation and characterization of germplasm is therefore becoming a priority, since this is exactly where the genetic variation necessary for future varietal improvement and for addressing future breeding challenges will be found [23].

A number of investigations aiming to characterize the phenotypic and genetic diversity of local collections of eggplant germplasm have been published in recent years [20], [24]–[27]. Hurtado et al. [28] have described both the phenotypic and DNA-based diversity present in a collection of entries sampled from three geographically well separated centers of diversity (China, Spain and Sri Lanka); their conclusion was that a combination of six plant traits was sufficient to assign the geographical origin of each entry, but that a similar level of discrimination was not possible using a set of 12 microsatellites; rather, the genotypic data suggested a measure of gene flow between the three centers of diversity. Furthermore, Meyer et al. [4], through historic and morphologic and molecular data based on nrITS sequences and AFLPs, made assumptions on phylogeographic relationships among candidate progenitors and Asian eggplant landraces and suggested a minimum of two domestications events which occurred in India and Southern China/SE Asia.

Here we describe a combined marker-based and morphological characterization of a wide set of “Occidental” and “Oriental” breeding lines, heritage varieties and selections from landraces. The objective was to assess the extent of genetic diversity that they contain, to illuminate the genetic relationship between “Occidental” and “Oriental” germplasm, and to provide criteria for the identification of a core germplasm collection. The genotypic data was represented by microsatellites, a class of genetic marker which thanks to its informativeness, reproducibility and co-dominant nature, has been widely employed for the analysis of plant genetic resources in many crops, including eggplant [22], [23], [25], [29].

Our results are of interest for conservation of genetic resources, their use in breeding programs, and contribute to the understanding of the evolutionary history of the species. Furthermore, in the context of our own research program, this data set sets the scene for an intended genotype/phenotype association study.

## Methods

### Permission

No specific permits were required for the described field studies, which took place in two experimental fields at the CRA-ORL in Montanaso Lombardo and CRA-ORA in Monsampolo del Tronto (Italy). These field plots were used by the authors of this paper affiliated to the fore mentioned institution (FC, LT, NA, TC, TS and GLR) for phenotypic characterization of the eggplant collection.

### Germplasm and Genotyping

The set of 238 entries was composed of 94 “Oriental” (Eastern - EA) types, hailing from China, Indo-China (specifying the region when known i.e. Thailand or Myanmar), Indonesia, India and Japan, and 139 “Occidental” (Western - WE) ones from Italy, France, Spain, Turkey and North Africa (Table 1). Genomic DNA was extracted from 2 g fresh young leaf harvested from three randomly chosen plants of each entry, using an E.Z.N.A.^T.M.^ Plant DNA mini kit (OMEGA bio-tek) according to the manufacturer’s protocol. The quality of each DNA sample was monitored by 0.8% agarose gel electrophoresis and its DNA concentration estimated spectrophotometrically (Beckman Coulter®, DU730). Each entry was then genotyped using a set of 24 microsatellite markers of known map location [30] and uniformly distributed across all 12 eggplant chromosomes (Table 2). Twenty-two were genomic SSRs [31]–[33]; while two (e.g. *ecm001* and *ecm023*) were EST-SSRs [31]. PCR amplification was performed according to [29], and successful amplicons were separated by denaturing 6% polyacrylamide gel electrophoresis on a LI-COR Gene ReadIR 4200 device, as described by Barchi et al. [30].

**Table 1:** The set of germplasm used for genotypic and phenotypic characterization.

ID	Accession Name	Origin	Areal	Structure subpopulations	Morphological grups
**AM_001**	**Dadali**	Indonesia	EA	B	1
**AM_004**	**Cima viola**	Italy	WE	A	1
**AM_005**	**Bianca ovale**	Italy	WE	B	2
**AM_010**	**1F5 (9)**	Breeding line	WE	A	2
**AM_011**	**Bianca Sicilia**	Italy	WE	B	2
**AM_013**	**CCR3**	Breeding line	WE	A	1
**AM_014**	**Mel**	Italy	WE	B	2
**AM_015**	**Luga 063**	Italy	WE	A	1
**AM_016**	**Prosperosa**	Italy	WE	B	3
**AM_018**	**Lunga Violetta Cinese**	China	EA	B	1
**AM_021**	**Tal 1/1**	Italy	WE	A	1
**AM_022**	**Angiò 4**	China	EA	B	1
**AM_023**	**BLK 1269**	Breeding line	WE	Admixtured	2
**AM_024**	**GIC/27-9**	Breeding line	WE	A	2
**AM_025**	**Tina**	Italy	WE	A	1
**AM_026**	**DR2**	Italy	WE	A	1
*AM_027*	*TBE80 D*	*Breeding line*	*WE*	*-*	*-*
**AM_028**	**TBE84 D**	Breeding line	WE	A	2
**AM_029**	**FanE13 D**	Breeding line	WE	A	2
**AM_030**	**FanE27 D**	Breeding line	WE	A	2
**AM_031**	**FanE63 D**	Breeding line	WE	A	2
**AM_032**	**SNL 534-11**	India	EA	B	3
**AM_033**	**SNL 533-8**	India	EA	B	3
**AM_034**	**SNL 600-1**	India	EA	B	2
**AM_035**	**Cin 01/24-6**	China	EA	B	2
**AM_036**	**Viola Cin-A-1**	China	EA	B	2
**AM_037**	**Violetta di toscana**	Italy	WE	B	3
**AM_038**	**Bellezza nera**	Italy	WE	A	2
**AM_040**	**Violetta di Metaponto**	Italy	WE	B	3
**AM_041**	**28-08/3 (23-09)**	Breeding line	WE	B	3
**AM_042**	**31-08/4 (25-09)**	Breeding line	WE	B	3
**AM_043**	**51-08/4 (29-09)**	Breeding line	WE	B	3
**AM_044**	**52-08/4 (30-09)**	Breeding line	WE	B	3
**AM_045**	**55-08/5 (31-09)**	Breeding line	WE	B	3
**AM_046**	**16-set**	Breeding line	WE	B	3
**AM_047**	**P621-08**	Breeding line	WE	B	3
**AM_048**	**P623-08**	Breeding line	WE	B	3
**AM_049**	**P645-08**	Breeding line	WE	B	3
**AM_050**	**P649-08**	Breeding line	WE	B	3
**AM_051**	**P612-08**	Breeding line	WExEA	B	3
**AM_052**	**P390**	Breeding line	WExEA	B	3
**AM_053**	**P328**	Breeding line	WExEA	B	3
**AM_054**	**P656-08**	Breeding line	WE	B	3
**AM_055**	**msp 73-08**	Breeding line	WE	A	2
**AM_056**	**S 1052-08**	Breeding line	WE	A	1
**AM_057**	**LI324/06**	Italy	WE	A	1
**AM_058**	**msp 36-08**	Italy	WE	A	1
**AM_059**	**msp 42-08**	Italy	WE	A	1
**AM_060**	**msp 30-08**	Italy	WE	A	1
**AM_062**	**msp 55-08**	Italy	WE	A	1
**AM_063**	**L422-08**	Italy	WE	A	1
**AM_064**	**L717-289**	Italy	WE	A	1
**AM_067**	**Uga**	Italy	WE	A	2
**AM_068**	**Tana**	Italy	WE	A	1
**AM_069**	**Bin 6**	Italy	WE	A	2
**AM_070**	**Floralba**	Italy	WE	A	2
**AM_071**	**Ind Min**	India	EA	B	1
**AM_072**	**SM5/2**	Breeding line	WE	A	2
**AM_073**	**SM5/13**	Breeding line	WE	A	2
**AM_074**	**SM5/22**	Breeding line	WE	A	1
**AM_076**	**S.Nicandro**	Italy	WE	Admixtured	2
*AM_085*	*LS96*	*Japan*	*EA*	*-*	*-*
**AM_086**	**LS 3805 minden**	Japan	EA	B	2
**AM_098**	**CIN6**	China	EA	B	3
**AM_099**	**CIN5**	China	EA	B	3
**AM_100**	**CIN7**	China	EA	B	3
**AM_102**	**CIN9**	China	EA	B	3
**AM_103**	**LS611**	Japan	EA	B	2
**AM_106**	**Naga-Ungu**	Indonesia	EA	B	1
*AM_107*	*N 286-1*	*India*	*EA*	*-*	*-*
*AM_109*	*N 285-B*	*India*	*EA*	*-*	*-*
*AM_110*	*N 24-6*	*India*	*EA*	*-*	*-*
*AM_111*	*N 243-3*	*India*	*EA*	*-*	*-*
*AM_112*	*N 286-A*	*India*	*EA*	*-*	*-*
*AM_113*	*N 321-14*	*India*	*EA*	*-*	*-*
**AM_114**	**N 258-4**	India	EA	B	1
*AM_116*	*N 220-A*	*India*	*EA*	*-*	*-*
*AM_119*	*N 324-A*	*India*	*EA*	*-*	*-*
**AM_121**	**Indom melanz**	Indonesia	EA	B	2
*AM_123*	*Pusa kranti*	*India*	*EA*	*-*	*-*
**AM_124**	**PI17**	Italy	WE	A	2
**AM_126**	**Almagro**	Spain	WE	Admixtured	2
**AM_127**	**Larga negra**	Spain	WE	A	1
**AM_128**	**Listada**	Spain	WE	A	2
**AM_129**	**Tolga**	Algeria	WE	A	2
**AM_133**	**Black Beauty**	Italy	WE	A	2
**AM_134**	**Viserba**	Italy	WE	A	1
**AM_135**	**Black Beauty**	Italy	WE	A	2
**AM_136**	**Tonda Violetta Firenze**	Italy	WE	B	2
**AM_137**	**Violetta Lunga Romagna**	Italy	WE	A	1
**AM_138**	**Barbentane**	France	WE	A	1
**AM_139**	**Lunga Marina**	Italy	WE	A	1
**AM_140**	**Tonda di Valence**	France	WE	B	3
**AM_141**	**Lunga Violetta Scura Cannellina**	Italy	WE	A	1
**AM_142**	**Tonda Black Beauty**	Italy	WE	A	2
**AM_143**	**Bellezza Nera**	Italy	WE	A	2
**AM_144**	**Lunga Violetta Napoli**	Italy	WE	A	1
*AM_145*	*Bianca Ovale*	*Italy*	*WE*	*-*	*-*
**AM_146**	**Black Beauty**	Italy	WE	A	2
**AM_147**	**Violetta Mostruosa NY**	Italy	WE	A	2
**AM_148**	**Slim Jim**	India	EA	B	1
**AM_149**	**Tonda Violetta Scura Valence**	France	WE	Admixtured	3
**AM_150**	**Grossissima Violetta Firenze**	Italy	WE	B	2
**AM_151**	**Violetta Lunga**	Italy	WE	A	1
**AM_152**	**Tonda Bianca**	Italy	WE	B	2
**AM_153**	**Prospera**	Italy	WE	A	3
**AM_155**	**Daejang**	China	EA	B	1
**AM_156**	**Buia**	Italy	WE	A	2
**AM_157**	**Baffa**	Italy	WE	A	2
**AM_158**	**Ank2**	India	EA	A	2
**AM_159**	**CN2**	China	EA	B	3
**AM_160**	**Dourga**	France	WE	A	2
**AM_162**	**Tunisia Baharia**	Italy	WE	B	3
**AM_163**	**Pusa Purple Cluster**	India	EA	B	1
**AM_167**	**Angio 3**	China	EA	B	1
**AM_168**	**Angio 5**	China	EA	B	2
**AM_169**	**Bianca striata verde**	Italy	WE	B	2
**AM_170**	**SM19/14**	Breeding line	WE	A	2
**AM_171**	**Palermitana**	Italy	WE	B	3
*AM_172*	*97-3 Drago volante*	*China*	*EA*	*-*	*-*
**AM_173**	**Pusa Purple Long**	India	EA	B	1
**AM_174**	**JM (Slim Jim)**	India	EA	B	1
**AM_175**	**Cannellina Sarnense**	Italy	WE	A	1
**AM_176**	**Sita**	Italy	WE	B	2
**AM_177**	**FiL withe**	Turchia	WE	B	2
**AM_178**	**Lunga napoli**	Italy	WE	A	1
**AM_179**	**1237/06**	Italy	WE	A	1
**AM_180**	**Listada Tacconi**	Italy	WE	A	2
**AM_181**	**Suraj(143)**	India	EA	Admixtured	2
**AM_182**	**Pusa Round**	India	EA	B	2
**AM_183**	**Chaojiuye Yuanquie**	China	EA	B	3
**AM_184**	**He Shanwang**	China	EA	B	3
**AM_185**	**Pp**	Indochinese Region	EA	B	3
*AM_186*	*Zf*	*Italy*	*WE*	*-*	*-*
**AM_187**	**Naveen**	India	EA	Admixtured	2
**AM_188**	**TAI 444**	Indochinese Region	EA	B	2
**AM_189**	**TAI 445**	Indochinese Region	EA	B	1
**AM_190**	**TAI 446**	Indochinese Region	EA	Admixtured	1
**AM_191**	**TAI 449**	China	EA	B	3
*AM_192*	*TAI 452*	*Indochinese Region*	*EA*	*-*	*-*
**AM_193**	**TAI 453**	Indochinese Region	EA	A	2
**AM_194**	**TAI 455**	Thailand	EA	B	2
**AM_195**	**TAI 456**	Myanmar	EA	B	2
**AM_196**	**TAI 457**	India	EA	Admixtured	2
**AM_197**	**TH 4438 Yad thip**	Thailand	EA	B	2
**AM_198**	**TH 6413 Raos**	Indonesia	EA	B	1
**AM_199**	**TAI 470**	Thailand	EA	B	2
**AM_200**	**TAI 475**	Thailand	EA	B	2
**AM_201**	**TAI 477**	Thailand	EA	Admixtured	2
**AM_202**	**TAI 480**	India	EA	B	2
**AM_203**	**TAI 481**	China	EA	B	1
**AM_204**	**TAI 483**	India	EA	Admixtured	1
**AM_205**	**TAI 484**	India	EA	Admixtured	1
**AM_206**	**7 CN**	China	EA	B	3
**AM_207**	**9 CN**	China	EA	B	3
**AM_208**	**17 CN**	China	EA	B	1
**AM_209**	**19 CN**	China	EA	Admixtured	-
**AM_210**	**67-3**	Breeding line	WExEA	B	3
**AM_211**	**305 E40**	Breeding line	WE	A	1
**AM_212**	**CGN17464 (PI 176759)**	Turkey	WE	A	2
**AM_213**	**CGN23345 (PI 169641)**	Turkey	WE	A	2
**AM_214**	**CGN18783 (Croisette)**	France	WE	B	1
**AM_215**	**CGN18531 (Patchem)**	Turkey	WE	B	1
**AM_216**	**CGN18510 (Bostan selection; PI 169666)**	Turkey	WE	A	-
**AM_217**	**CGN17449 (Topak; PI 175917)**	Turkey	WE	A	2
**AM_218**	**CGN17451 (Dolmalik; PI 176758)**	Turkey	WE	A	2
*AM_219*	*CGN17571 (PI 169641)*	*Turkey*	*WE*	*-*	*-*
*AM_220*	*CGN17574 (PI 169643)*	*Turkey*	*WE*	*-*	*-*
**AM_221**	**CGN17579 (PI 169648)**	Turkey	WE	A	-
**AM_222**	**CGN23346 (Topatan; PI 169649)**	Turkey	WE	Admixtured	2
*AM_223*	*CGN23347 (PI 169650)*	*Turkey*	*WE*	*-*	*-*
**AM_224**	**CGN17581 (PI 169651)**	Turkey	WE	A	1
**AM_225**	**CGN17564 (PI 166994)**	Turkey	WE	A	-
**AM_226**	**CGN23341 (Kemer Patlican; PI 167101)**	Turkey	WE	A	-
**AM_227**	**CGN23342 (Patlican; PI 167209)**	Turkey	WE	A	-
**AM_228**	**CGN23343 (PI 167328)**	Turkey	WE	A	1
*AM_229*	*CGN17568 (Yuvorlak Patlican; PI 167373)*	*Turkey*	*WE*	*-*	*-*
**AM_230**	**CGN23344 (Bostan; PI 169639)**	Turkey	WE	A	2
**AM_231**	**CGN18591 (PI 171847)**	Turkey	WE	A	2
**AM_232**	**CGN18595 (PI 171852)**	Turkey	WE	A	1
**AM_233**	**CGN18779 (De Barbentane)**	France	WE	A	1
**AM_234**	**CGN23309 (Dolg; PI 358232)**	Macedonia	WE	A	1
**AM_235**	**CGN18484 (Morska Pata; PI 358242)**	Macedonia	WE	B	1
**AM_236**	**CGN18782 (Violette Longue Hative)**	France	WE	A	1
*AM_237*	*CGN18512 (Violette Noire d`Orient)*	*France*	*WE*	*-*	*-*
**AM_238**	**CGN17453 (Yesilkoy 27)**	Turkey	WE	A	1
*AM_239*	*CGN17582 (Alacali; PI 169652)*	*Turkey*	*WE*	*-*	*-*
**AM_240**	**CGN18578 (Kemer; PI 169655)**	Turkey	WE	Admixtured	-
**AM_241**	**CGN23348 (PI 169658)**	Turkey	WE	A	1
**AM_242**	**CGN18581 (PI 169660)**	Turkey	WE	Admixtured	-
**AM_243**	**CGN18585 (PI 169663)**	Turkey	WE	A	1
*AM_244*	*CGN23349 (PI 169663)*	*Turkey*	*WE*	*-*	*-*
*AM_245*	*CGN18601 (PI 173106)*	*Turkey*	*WE*	*-*	*-*
*AM_246*	*CGN18602 (PI 173107)*	*Turkey*	*WE*	*-*	*-*
*AM_247*	*CGN18605 (PI 173807)*	*Turkey*	*WE*	*-*	*-*
**AM_248**	**CGN18610 (Bostan; PI 174361)**	Turkey	WE	Admixtured	-
**AM_249**	**CGN23351 (PI 174362)**	Turkey	WE	A	2
*AM_250*	*CGN23352 (PI 174367)*	*Turkey*	*WE*	*-*	*-*
**AM_251**	**CGN24467 (Berenjena Listada)**	Spain	WE	A	2
**AM_252**	**CGN18505 (Berenjena Redonda)**	Spain	WE	Admixtured	3
**AM_253**	**CGN24468 (Caminal)**	France	WE	A	1
*AM_254*	*CGN17472 (Redonda Negra Lisa)*	*Spain*	*WE*	*-*	*-*
*AM_255*	*CGN23318 (Larga Negra)*	*Spain*	*WE*	*-*	*-*
*AM_256*	*CGN18511 (Indonesische Aubergine)*	*Indonesia*	*EA*	*-*	*-*
**AM_257**	**CGN18776 (Longue Hative)**	France	WE	A	1
**AM_258**	**CGN17456 (Monda)**	France	WE	Admixtured	2
**AM_259**	**CGN23315 (Ronde de Valence)**	France	WE	Admixtured	3
**AM_260**	**CGN17479 (Semiredonda Jaspeada)**	Spain	WE	A	2
*AM_261*	*CGN23323 (PI 120770)*	*Turkey*	*WE*	*-*	*-*
**AM_262**	**CGN23772**	Nigeria	WE	Admixtured	2
**AM_263**	**Violetta precoce**	Italy	WE	Admixtured	-
**AM_264**	**Mezza Lunga Violetta**	Italy	WE	A	1
**AM_265**	**Lunghissima Precoce Violetta**	Italy	WE	A	1
**AM_266**	**Dingaras**	China	EA	B	1
*AM_267*	*Bioleta*	*Spain*	*WE*	*-*	*-*
**AM_268**	**L 129**	Indonesia	EA	B	1
**AM_269**	**Talindo**	Indonesia	EA	B	1
**AM_271**	**DS1**	Breeding line	WE	A	2
**AM_273**	**DS2**	Breeding line	WE	A	2
**AM_274**	**DS4**	Breeding line	WE	A	2
**AM_275**	**1 CAAS**	China	EA	B	3
*AM_276*	*2 CAAS*	*China*	*EA*	*-*	*-*
*AM_277*	*3 CAAS*	*China*	*EA*	*-*	*-*
**AM_278**	**4 CAAS**	China	EA	B	3
**AM_279**	**5 CAAS**	China	EA	B	3
*AM_280*	*6 CAAS*	*China*	*EA*	*-*	*-*
*AM_281*	*7 CAAS*	*China*	*EA*	*-*	*-*
*AM_282*	*8 CAAS*	*China*	*EA*	*-*	*-*
*AM_283*	*9 CAAS*	*China*	*EA*	*-*	*-*
**AM_284**	**10 CAAS**	China	EA	A	1
**AM_285**	**11 CAAS**	China	EA	B	1
*AM_287*	*13 CAAS*	*China*	*EA*	*-*	*-*
**AM_288**	**14 CAAS**	China	EA	A	2
**AM_289**	**15 CAAS**	China	EA	B	3
**AM_290**	**16 CAAS**	China	EA	B	3
**AM_291**	**17 CAAS**	China	EA	B	3
**AM_292**	**18 CAAS**	China	EA	B	1
**AM_293**	**19 CAAS**	China	EA	B	1

Those shown in *italics* refer to entries retaining a level of heterozygosity >10%, and those shown underlined produced off-types with respect to plant and/or fruit type. Retained entries are shown in **bold**.

**Table 2:** The 24 microsatellite loci used for genotyping.

Marker	Chromosome	Position (cM)	Alleles	Rare alleles	PIC
*CSM 31*	E01	107.4	12	2	0.83
*ecm001*	E01	77.7	7	2	0.73
*emh21J12*	E01	91.8	11	5	0.76
*emf01G17*	E02	35.4	10	5	0.65
*EM 133*	E02	11.6	6	4	0.24
*emg11I03*	E03	6.0	6	2	0.77
*emj03A17*	E03	34.3	3	1	0.38
*emf01K16*	E04	0.0	4	0	0.63
*EM 117*	E04	47.6	6	1	0.76
*emf01A06*	E05	64.3	4	1	0.45
*EM 146*	E05	50.6	7	2	0.68
*CSM 7*	E06	35.6	3	0	0.48
*CSM 19*	E07	0.0	4	1	0.55
*CSM 69*	E07	73.4	2	0	0.48
*ecm023*	E08	13.9	2	0	0.35
*emi03M03*	E08	13.5	3	0	0.45
*CSM 54*	E09	16.1	7	1	0.66
*eme03B08*	E10	46.5	6	0	0.74
*emf11F07*	E10	53.7	7	1	0.75
*emf21K08*	E11	0.0	9	3	0.63
*EM 080*	E11	25.6	2	0	0.35
*CSM 29*	E12	82.3	6	1	0.67
*CSM 73*	E12	0.0	5	1	0.69
*emb01O01*	E12	43.9	8	1	0.77
total			140	34	0.60

Two of the assays (*ecm001* and *ecm023*) were designed from EST sequence [29], while the others were designed from genomic sequence [29]–[31]. The chromosome location, the number of total and rare alleles detected and the PIC values are listed.

### Phenotypic Characterization

The entries were each scored for 19 plant, leaf, flower and fruit traits (Table 3), included among the European Cooperative Program for Plant Genetic Resource Solanaceae and/or the International Board for Plant Genetic Resources eggplant descriptors. Peel color was measured using a Chroma-meter Minolta CR-400 on the basis of the three Hunter color coordinates (L*, a* and b*), and represented the average of three randomly chosen portions of each fruit. The measurements were reduced to a single variate by calculating the Euclidean distance from white (L* = 100, a* = 0, b* = 0), following Prohens et al. [34].

**Table 3:** Traits analysed to generate the phenotypic data set.

Trait	Code	Evaluation method
Peel color	pcol	L*a*b* color coordinates
Peel glossiness	pglo	Scale from 0 (high opacity) to 3 (high glossiness)
Fruit curvature	fcur	Scale: 1 (no curvature), 5 (curved), 9 (U shaped)
Fruit weight	fw	Grams
Fruit length	fl	Centimeters (from the base of the calyx to the tip of the fruit)
Fruit diameter max	fdmax	Centimeters
Fruit diameter max position	fdmaxp	Scale from 1 (close to the calyx) to 8 (close to the apex)
Fruit shape	fs	fl/frdmax
Flesh fruit firmness	firm	Scale from 1 (very loose) to 9 (very dense)
Leaf hairiness	lha	Scale from 0 (no hairiness) to 5 (highly hairiness)
Adaxial leaf lamina anthocyanin	adlan	Scale from 0 (green) to 5 (complete purple coloration)
Stem anthocyanin	stean	Scale from 0 (green) to 5 (complete purple coloration)
Calyx coverage of the fruit	cacov	Scale from 1 (<10% of the fruit length) to 5 (>50%)
Fruit calix prickliness	fcpri	Scale from 0 (no prickles) to 9 (high prickliness)
Leaf prickliness	lepri	Scale from 0 (no prickles) to 5 (high prickliness)
Plant growth habit	hab	Scale from 1 (upright ) to 9 (prostrate)
Inflorescence flowers	inflw	Number of flowers for inflorescence
Flowering abundance	flwab	Number of flowers on the plant, scale from 1 (very few) to 5 (many)
Flowering time	flwt	Number of days from seedling emergence after sowing when at least 50% of the plants have its first flower opened

The germplasm was grown in two locations: Montanaso Lombardo [ML]: 45 20′N, 9 26′E, and Monsampolo del Tronto [MT]: 42 53′N; 13 47′E in each of 2010 and 2011. For each field experiment, six plants per entry were planted in two completely randomized blocks with a 1 m inter-row and a 0.8 m inter-plant within row distance. Standard crop management practices were applied.

### Analysis of Marker Data

The scoring of microsatellite data was imported into Past 2.08 software [35]. and pair-wise similarity coefficients [36] were computed. Alleles occurring at a frequency ≤1% were considered as rare. A principal co-ordinate (PCO) analysis was carried out to display the multi-dimensional relationships between entries. The polymorphic information content (PIC) of each microsatellite locus was evaluated by applying the following equation, as suggested by Anderson [37]: PIC = 1-∑ *P* ^2^_ij,_ where *P*_ij_ represented the frequency of the *j* ^th^ allele at the *i* ^th^ microsatellite locus and the summation was extended over *n* alleles. The Bayesian-based model procedure implemented by STRUCTURE 2.3 software [38] was used to determine population structure; *K* values from 1 to 15 were tested. A burn-in period of 50,000 and 100,000 rounds from ten independent simulations were used to assess the population structure. The most likely number of sub-groups present was based on minimizing Δ*K* [39]. Population structure was also characterized using the fixation index statistics provided within the STRUCTURE 2.3 package. To identify the minimum number of entries required to retain 100% of the allelic diversity present in the full germplasm set, the M strategy suggested by Schoen and Brown [40], as implemented in the MSTRAT software [41], was used. The number of iterations per MSTRAT run was 30, and the number of repetitions for core sampling was 20. The entries most frequently represented across the 30 replicates formed the core collection. The efficiency of the strategy was assessed by comparing the total number of alleles captured using MSTRAT in samples of increasing size to the number of alleles captured in randomly chosen collections of equal size (ten independent samplings).

### Analysis of Morphological Data

The morphological data were treated as adjusted entry means (best linear unbiased predictors, BLUPs). The variance components were determined using the restricted maximum likehood (REML) method applying the mixed linear model *p_ijsb_ = l_j_+y_s_+g_i_+r_bjr_+e_ijs_*, where *p_ijsb_* was the phenotypic value of the *b^th^* replicate of *i^th^* entry at the *j^th^* location in the *s^th^* year, *l_j_* the contribution of the *j^th^* location, *y_s_* the contribution of the s*^th^* year, *g_i_* the contribution of the *i^th^* entry, *r_bjs_* the contribution of the *b^th^* replicate within the *j^th^* location in the *s^th^* year, and *e_ijs_* the residual error. A principal component analysis (PCA) was carried out to determine which traits acted as the prime discriminators between entries. Common components coefficients, eigenvalues and the proportion of the total variance expressed by each single trait were calculated. The Scree plot was used to select the components most relevant for the ordination analysis. Correlations between traits and each principal component were calculated, and those ones having an absolute value >0.5 were considered relevant for the trait’s determination [42]. An hierarchical clustering on principal components (HCPC) analysis was performed to define a set of clusters based on phenotypic traits. The cluster analysis was performed only on the most significant PCA components, with the remaining minor ones considered to represent noise [43]. Only dimensions having an eigenvalue >1 (Kaiser’s method) were considered. The hierarchical clustering was performed according to the Ward criterion, based on variance evaluation (inertia) as well as on the principal component method. In order to define the appropriate number of clusters, both the overall shape of the tree and the bar plot of the gain in inertia were considered. The presence of a difference between the clusters for each trait was tested using a Kruskal-Wallis analysis of variance, and a Nemenyi *post hoc* test was performed on traits displaying differences to identify which groups were involved. The above analyses were implemented with R software [44]. A co-phenetic correlation between the genotypic and phenotypic data matrices was calculated, and tested using the Mantel [45] method, including 5,000 permutation as implemented in Past 2.08 software [35].

## Results

### Microsatellite Diversity

Across the set of 238 eggplant entries, 140 alleles were identified at the 24 microsatellite loci (average 5.8 per locus) (Table 2), and each entry had a distinct genotype. The loci varied in terms of the number of alleles present from two (*EM 080*, *ecm023* and *CSM 69*) to 12 (*CSM 31*), while their PICs ranged from 0.24 (*EM 133*) to 0.83 (*CSM 31*), with a mean of 0.60. There were 34 rare alleles, of which 14 were only found in the “Oriental” germplasm and the other 20 only in the “Occidental” germplasm. A residual level of heterozygosity >10% was present in 38 entries, and as a result, these entries were not considered for phenotyping (Table 1). The average Dice similarity coefficient for the 200 fixed lines was 0.32.

**Figure 1:**
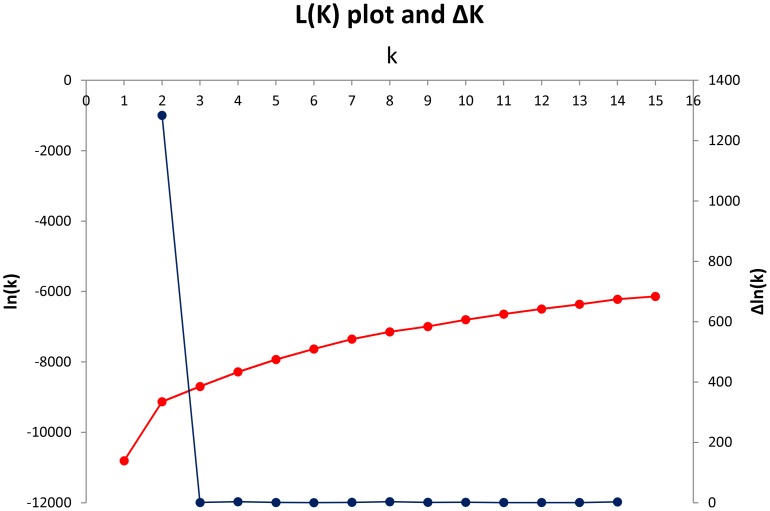
STRUCTURE analysis. (*K*) and Δ*K* plots derived from the genotypic data. The germplasm set forms two distinct sub-groups, with a small number of entries being intermediate.

**Figure 2:**
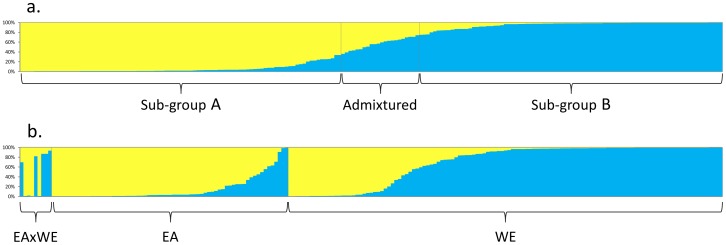
STRUCTURE output at *K* = 2. Each entry is represented by a horizontal line representing subgroup 1 (yellow) and subgroup 2 (blue). a) Entries ordered according to their subgroup membership. b) Entries ordered according to their geographical origin: WE: “Occidental”, EA: “Oriental”.

STRUCTURE analysis with different K-levels (1–15) were assayed and *K* value for 2 was optimal (Figure 1). According to output of structure analysis (Figure 2a) each accession was assigned to a sub-group (A or B) when its level of membership was higher than 70% (Table 1). Sub-group A comprised 89 entries and sub-group B 90 entries, with the remaining 21 defined as admixed. The fixation index was 0.30 for sub-group A and 0.18 for sub-group B, indicating that a certain amount of structuration was still present within each of them. Applying the M method showed that the minimal set sufficient to capture all 106 non-rare alleles was 16 (“sub-16”), while the size of set required to capture all 140 alleles was 48 (“sub-48”). Random sampling was less efficient at retaining alleles, since randomly chosen sets of 16 entries captured only 96.5 alleles on average, and randomly chosen sets of 48 only 109.3 alleles.

### Morphological Variation

**Figure 3:**
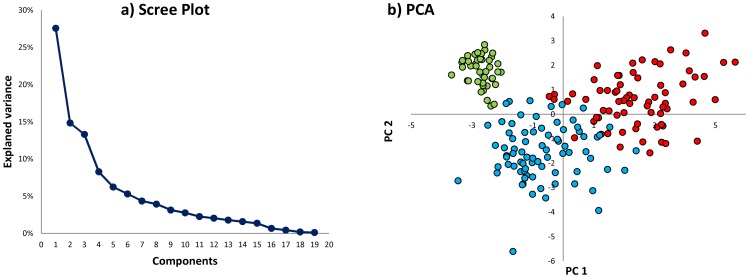
HCPC analysis, based on the leading six PC’s (eigenvalues >1). a) Scree plot showing the proportion of variance explained by each PC. b) PCA based on the leading two PC’s. Entries belonging to each morphological group marked by a different color (red: group 1, blue: group 2, green: group 3).

**Figure 4:**
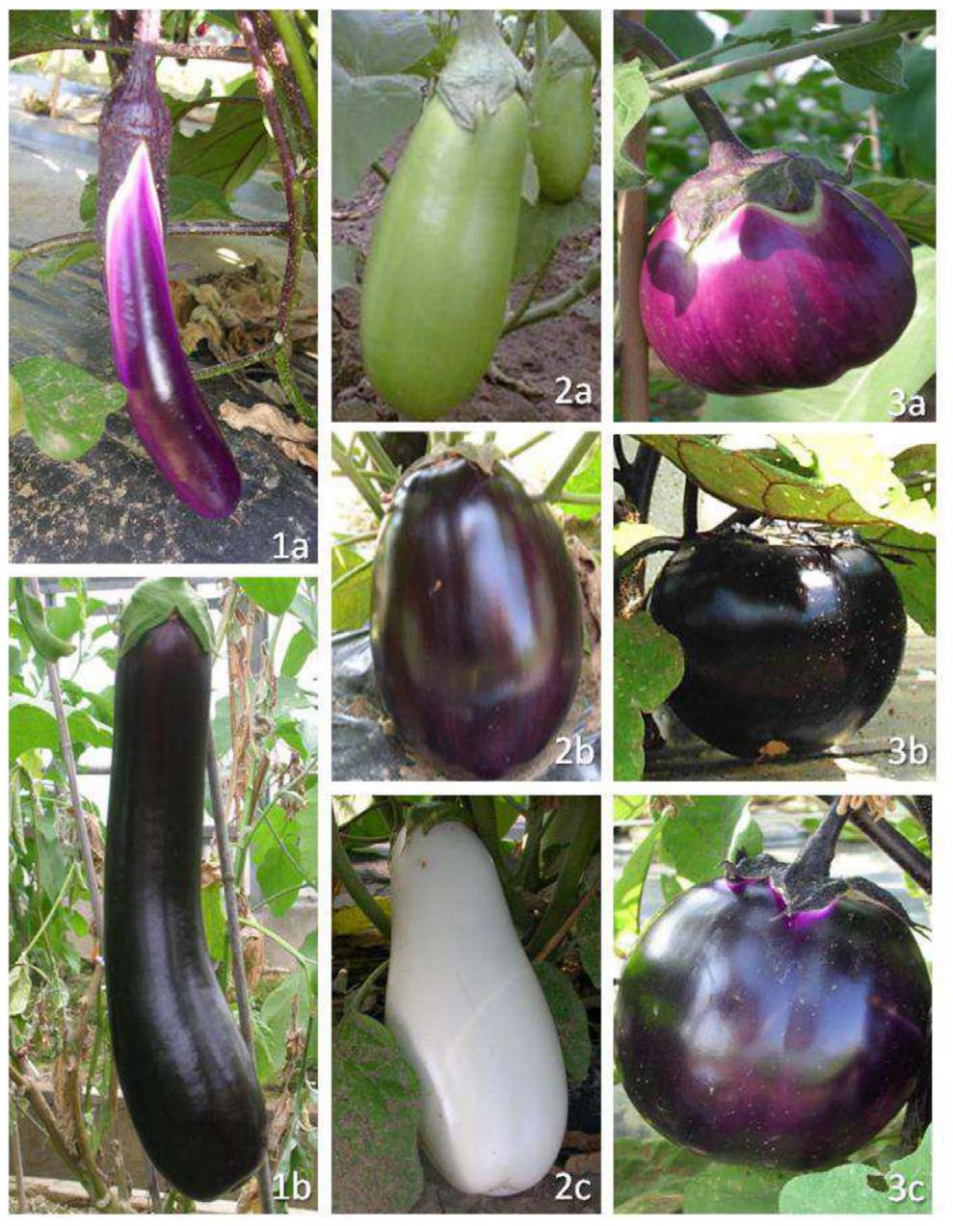
Fruits of accessions belonging to the three main morphological groups. Group 1∶1a = AM 269-Talindo; 1b = AM 026-Dr2; Group 2∶2a = AM 168-Angio 5; 2b = AM 031-FantE63D; 2c = AM 160-Dourga; Group 3∶3a = AM 037-Violetta di Toscana; 3b = AM 291∶17CAAS; 3c = AM 210-67/3.

Among the 200 fixed lines, off types with respect to plant and/or fruit traits were present in nine, so the full phenotyping set was further reduced to 191 entries (Table 1). The phenotypic performance of these entries is reported in Table 4. The most variable traits were fruit size, weight, shape and curvature, along with peel color. The PCA scree plot showed that 55.7% of the overall phenotypic variation was captured by the first three principal components (PC’s) (Figure 3a). The correlation coefficients for each trait with each of these three PC’s, along with the associated eigenvalues and proportions of the total variance explained, are detailed in Table 5. The first PC explained 27.6% of the variance and was positively correlated with fruit length (+0.89), shape (+0.92) and curvature (+0.89), as well as the distance of the widest part of the fruit from the fruit apex (+0.76); it was simultaneously negatively correlated with the maximum diameter of the fruit (−0.91), fruit weight (−0.76) and flesh firmness (−0.74). PC2 explained 14.8% of the variance, and was positively correlated with the anthocyanin content of the stem (+0.86) and leaf (+0.76), and with the intensity of the peel color (+0.52). PC3 explained 13.3% of the variance, and was positively correlated with late flowering (+0.55) and negatively with flowering abundance (−0.71) and the presence of a prostrate growth habit (−0.51). The subsequent HCPC analysis was based on the leading six PC’s (with eigenvalues >1), which together explained 75.4% of the variance. Three main morphological groups were identified (Figure 3b) and the differences between these groups are detailed in Table 4. Entries belonging to the group 1 (Figure 3b, area I/II) produced long, light (average weight ∼150 g) and curved fruits, the flesh of which was of only limited firmness and the peel was purple; the anthocyanin content of both the leaves and stems was intermediate, plant habit was erect and the plants formed many flowers per inflorescence. The entries within group 2 (Figure 3b, area II/III) produced oblong-shaped fruits of average weight of ∼250 g; peel color was white, green or light violet, the plants were semi-erect and the leaves and stems contained little anthocyanin. Finally, group 3 entries (Figure 3b, area IV) produced rounded, heavy (average weight ∼400 g) and dark purple colored fruits; calyx and leaf prickliness was largely absent, the anthocyanin content of both the leaves and stems was high and the number of flowers per inflorescence was low. Examples of fruits belonging to the three morphological groups are reported in Figure 4.

**Table 4:** The distribution of trait-by-trait performance across the 191entries phenotyped (the ones not showing residual heterozygosity as well as phenotypic variation), and the statistical significance of the three morphology-based groups identified.

Trait code	Average	St. Dev.	ANOVAbetween groups	Post hoc test			Group average		
				1 vs 2	1 vs 3	2 vs 3	1	2	3
pcol	74.57	15.60	***	NS	NS	***	79.89	69.21	81.61
pglo	2.37	0.73	***	NS	**	***	2.36	2.19	2.76
fcur	2.08	1.38	***	***	***	NS	3.95	1.61	1.03
fw	256.97	122.26	***	***	***	***	148.28	243.99	395.11
fl	14.39	5.53	***	***	***	**	21.43	13.16	9.32
fdmax	7.39	2.59	***	***	***	***	4.53	7.28	10.74
fdmaxp	5.84	0.72	***	**	***	***	6.48	5.84	5.05
fs	2.49	1.88	***	***	***	***	5.03	1.95	0.88
firm	5.33	1.62	***	**	***	***	3.58	5.72	6.38
lha	1.91	1.15	NS	NS	NS	NS	1.58	2.05	1.98
adlan	1.37	1.49	***	***	**	***	1.79	0.42	3.13
stean	2.80	1.84	***	***	**	***	3.59	1.53	4.95
cacov	2.44	0.74	NS	NS	NS	NS	2.29	2.52	2.28
fcpri	1.65	1.83	NS	NS	NS	NS	1.35	1.88	1.16
lepri	0.43	0.65	***	NS	*	*	0.46	0.51	0.20
hab	3.83	1.62	***	***	NS	***	2.94	4.47	3.41
inflw	1.94	0.95	***	NS	*	***	2.20	1.99	1.52
flwab	2.24	0.76	NS	NS	NS	NS	2.25	2.25	2.32
flwt	86.43	5.97	NS	NS	NS	NS	85.90	86.48	85.98

* P<0.05

** P<0.01

*** P<0.001

**Table 5:** Correlation coefficients between each trait and the leading three PC’s, along with the associated eigenvalues and proportions of the overall variance explained.

Trait code	Common principal component coefficient		
	First	Second	Third
pcol	−0.01	0.52	0.45
pglo	−0.14	0.34	0.49
fcur	0.89	0.20	0.12
fw	−0.76	0.09	0.44
fl	0.89	0.06	0.30
fdmax	−0.91	0.11	0.23
fdmaxp	0.76	−0.19	0.20
fs	0.92	0.15	0.08
firm	−0.74	−0.10	−0.09
lha	−0.28	−0.20	0.04
adlan	−0.22	0.76	−0.05
stean	−0.14	0.86	0.03
cacov	−0.05	−0.44	0.42
fcpri	−0.09	−0.50	0.42
lepri	0.04	−0.48	0.30
hab	−0.24	−0.44	−0.51
inflw	0.20	−0.09	−0.39
flwab	0.01	0.15	−0.71
flwt	−0.05	−0.16	0.55
**Eigenvalue**	5.24	2.82	2.53
**Variability %**	27.6%	14.8%	13.3%
**Accumulated variability %**	27.6%	42.4%	55.7%

### The Relationship between Phenotype, Genotype and Geographical Origin

**Figure 5:**
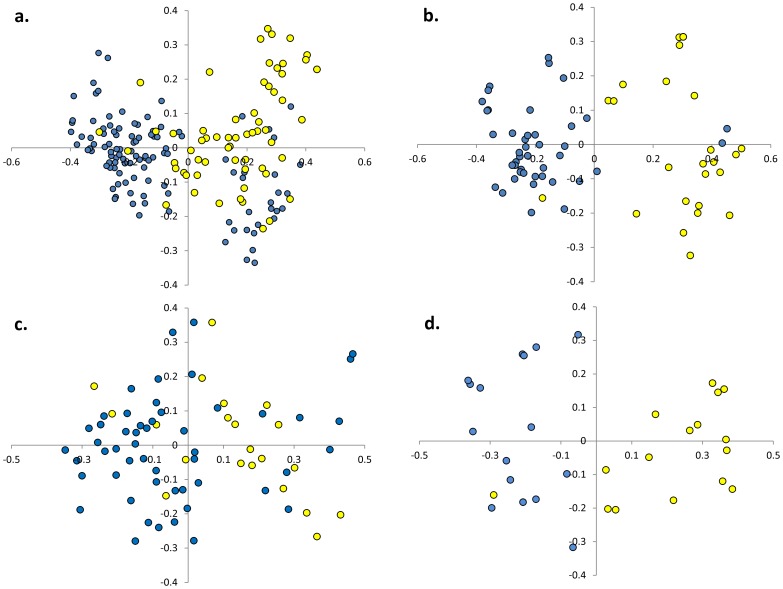
PCA based on geographical origin (blue: “Occidental”; yellow: “Oriental”). a) The full germplasm set, and entries within b) morphological group 1, c) morphological group 2, d) morphological group 3.

**Figure 6:**
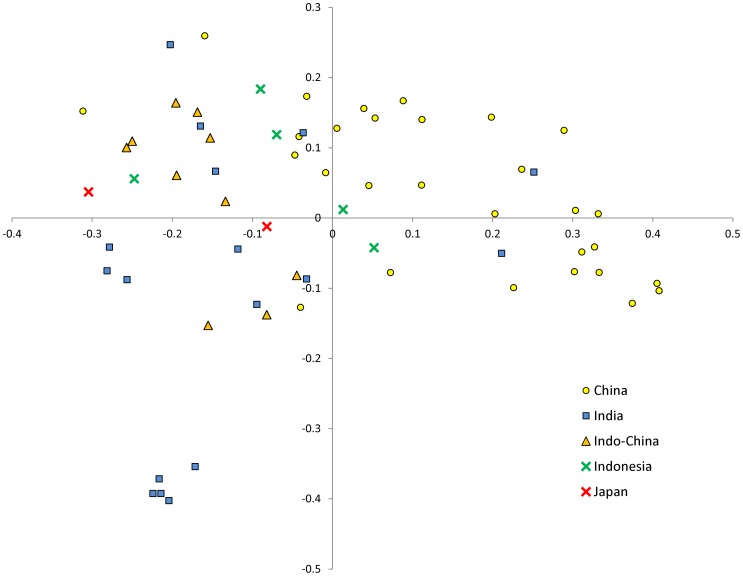
PCA based on geographical origin showing the clustering of the “Oriental” entries with their country of origin. The accessions from Myanmar and Thailand were classified as Indochinese region.

All three morphological groups were represented in both the “Occidental” and the “Oriental” germplasm (Table 1). Group 1 types comprised 39% of the “Occidental” set, group 2 types comprised 45% and group 3 types comprised 16%, while the respective proportions for the “Oriental” germplasm were 35%, 30% and 35%. According to a Mantel test, there was only a weak correlation (0.23) between the phenotypic and the genotypic data sets. A PCO analysis of the microsatellite data showed that entries belonging to each of the three morphological groups were scattered across the whole PC space (not shown). However, there was a perceptible relationship between genotype and geographical origin, since the PCO analysis showed that most of the “Oriental” entries mapped to the right hand section of the PC plane and the most of the “Occidental” ones to the left hand section (Figure 5a). A similar relationship was revealed by STRUCTURE analysis, once the entries were grouped according to their geographical provenance (Figure 2b). Some 65% of the “Oriental” entries were captured by sub-group A, as were 96% of the “Occidental” entries by sub-group B. The average pair-wise genetic similarity between the “Oriental” and “Occidental” entries was just 0.31, highlighting the extent of genetic differentiation between these two sets of germplasm. In contrast, the average pair-wise genetic similarity between entries within a geographical group was 0.44 (“Oriental”) and 0.46 (“Occidental”); although the entries within these groups were more similar to one another than were the entries between the groups, there still remains a considerable amount of within group genetic variation in both regions. When the PCO was applied to entries sorted by morphological group, the “Occidental” *vs* “Oriental” distinction was retained (Figure 5b–d), although the relationship was weakest for the group 2 types (Figure 5c). A PCO analysis of the genotypic data performed within each of the two areas showed a clustering of Chinese germplasm within the “Oriental” germplasm (right hand section of Figure 6), and similarly of India/Burma entries (left hand side). No equivalent clustering was evident in the “Occidental” germplasm (data not shown).

## Discussion

Eggplant varieties/landraces are morphologically, physiologically and biochemically highly variable, but the progressive dominance of elite F_1_ hybrids in commercial cultivation presents a threat of genetic erosion, which in the longer term may well have negative implications by narrowing the source of useful genes exploitable in breeding programmes [26]. Previous attempts to characterize diversity have been restricted to a limited number of local varieties/landraces; [1], [24], [31], [33], [46]–[51]. Two recent studies have focused on 52 accessions identified from three secondary centers of origin of the crop [23] or 115 genotypes from Asian landraces and some wild relatives [4]. Here we have presented a phenotypic (19 traits) and genotypic (24 microsatellite loci) survey of a large germplasm collection originating from both Asia and the Mediterranean Basin, and representing a mixture of breeding lines, heritage and current varieties and landrace selections.

*S. melongena* is a largely autogamous species, so that the expectation is that most heritage and commercial varieties should be highly homozygous. The microsatellite-based genotyping uncovered some residual heterozygosity in the germplasm set, which led to the discarding of some 16% of the entries. A further 4% produced phenotypic off-types, presumably also reflecting the presence of residual heterozygosity (although it may also reflect admixture), leaving a panel of 191 true-breeding, largely homozygous entries. There was ample variation with respect to both plant and fruit traits within both “Oriental” and “Occidental” entries, and it was possible to derive a set of just three morphology-based PC’s to explain over half of the phenotypic variance displayed by the full set of 19 traits (Figure 3a). Both the leading two PC’s were correlated with fruit shape and dimension, as well as with anthocyanin content, as has previously been reported for a set of Spanish varieties [24]. As for many other crops [52], the fruit has been a major target of anthropogenic selection. Anthocyanin content, a trait acquired during domestication (since the eggplant’s putative ancestor *S. insanum* produces green fruit [1]), may have been under both indirect selection, based on its involvement in tolerance to a number of environmental stresses, and direct selection, due to cultural preferences towards pigmented fruits [53], [54].

The HCPC analysis identified three main groups (Figure 3b). The first one included genotypes producing elongated fruits, with a mean fs (fruit length/fruit maximum diameter) around 5.05 (Table 4). This group corresponds to the one previously detected within the eggplant Spanish germplasm (fs >2) [55], [56] as well as to the fruit typology defined *var. serpentinum* (long and slender fruit) identified by Choudhury [57]within the Indian germplasm. The second and the third morphological groups, with a mean fs of 1.95 and 0.98 respectively, are classified together in the fruit typology *var. esculenta* (round or egg-shaped fruit) identified by Choudhury [57], while they are separately identified as genotypes bearing semi-long fruits (with a fs >1.2 and <2) and round fruits (with a fs ∼1) by Prohens et al. [56] and Nuez et al. [55].The three morphological groups cut across the “Oriental” *vs* “Occidental” divide. In contrast, the conclusion of Hurtado et al. [23], based on an analysis of entries originating from China, Spain and Sri Lanka, was that a number of traits could be associated with the geographical origin of the material. The apparent discrepancy can be explained by either the difference in size of the two germplasm sets (52 *vs* 191) and/or by the somewhat different set of traits assessed in the two studies. Germplasm sets which capture a wide range of phenotypic variation tend to form many clusters when many traits are scored and few when only few traits are scored [58], [59]. The present HCPC analysis identified three distinct and robust groups, based on variation in 14 out of the full set of 19 traits recorded. Nevertheless, there was only a weak correlation between phenotype and molecular fingerprinting, an experience also recorded by Hurtado et al. [23]; in contrast, both the Munoz-Falcon et al. [22] and Prohens et al. [24] studies showed a reasonable level of phenotype/genotype correlation, probably because both focused on germplasm of rather limited diversity. The relationship between rates of phenotypic evolution and genetic change has been a matter of debate, but the rate of molecular evolution has been by many authors considered to be not strictly associated to the rate of morphological change, as only a tiny portion of the genome is directly responsible for the measurable phenotypic changes [60]. The two types of markers follow different evolutionary paths and provide complementary information contributing in understanding both evolutionary history and identifying the most suitable strategy for germplasm management [61].

When the STRUCTURE analysis was based on geographical provenance (Figure 2b), most of the “Oriental” entries fell into one cluster and most of the “Occidental” ones into another. The PCO analysis of the microsatellite data also differentiated clearly between the two provenances. A clustering in relation to provenance was also detected when PCO analysis was separately performed within each of the three main morphological groups (Figure 5b, 4c, 4d). This highlights that a molecular differentiation is detectable also between Oriental and Occidental entries with similar phenotypic traits.

When the PCO analysis was applied to just the “Occidental” entries, no evidence of any correlation between provenance and genetic relatedness was found (data not shown), suggesting that this gene pool has experienced extensive exchange of breeding materials. The picture is rather different for the “Oriental” gene pool (figure 6), in which a trend of clustering was detected and most of the genotypes from the Indian, Indo-Chinese and Indonesian regions grouped together and separately from the Chinese ones. Recent studies highlight that the modern eggplant evolved from the species *S. insanum* [2], and it has been generally assumed that it was domesticated in Indian subcontinent[3], ,possibly in Rajasthan region [5]. The distinct genetic content of Chinese germplasm uncovered in the present analysis supports the alternate idea proposed by Wang et al. [6], Ali et al. [20] and Meyer et al. [4], that a secondary site of domestication also developed in China. Multiple, rather than single, domestication events seem to apply for a number of crops [66]. The introduction of the eggplant to the Mediterranean Basin by the Arabs would have generated a temporary bottleneck in genetic diversity [54] but still maintaining a rather large share of variability [67] and which was alleviated by subsequent selection, *de novo* mutations and recombination events as well as adaptation to different environments [68].

This, despite some movement of germplasm across the Asian and Mediterranean countries occurred over time, justify the genetic differentiation we detected between genotypes from the two geographical areas.

Plant germplasm management is pivotal for providing the plant scientist with sufficient genetically, well-characterized material for research and crop improvement. To this purpose the development of genetic core collections helps to provide a reduced set of accessions, in terms of entry number but not in terms of allelic coverage, that are feasible to study and handle. A critical examination of the various methods used to evaluate the quality of core collections suggests a lack of consensus regarding the optimal selection criteria to be applied [69]. Here, the retention of about 25% of the collection was required to capture all the microsatellite alleles present in the full set; the need for such a large proportion is a consequence of the species’ high level of homozygosity, since a heterozygote by definition harbors two alleles, whereas a homozygote only harbors one. Similar proportions have to be retained in both *Arabidopsis thaliana* (18%, [70]) and *Medicago truncatula* (31% [71]), while a heterozygous species, such as grapevine, required a retention level as low as 4% [72].

Some of the phenotypic diversity identified in the present germplasm would doubtless be of interest to conventional eggplant improvement programs. However, the application of more efficient selection programs requires the understanding of the genetic basis of key agronomic traits, via the development of linkage maps and quantitative trait locus (QTL) analysis. Thus, for example, Miyatake et al. [73] have defined the genetic control of parthenocarpy, while Barchi et al. [74] were able to identify a number of QTL underlying anthocyanin pigmentation. The association mapping approach has been proposed as an alternative platform to conventional linkage analysis for QTL detection [75]. The concept relies on analyzing a large set of germplasm in which there is a substantial level of morphological and genetic diversity built up by a history of recombination and re-assortment and whose population structure has been carefully assessed. One of the intentions of the present study is to identify such a population in eggplant, and the present analysis has provided important information regarding both the potential diversity available in the species and the likely sources of population structure. The data set as a whole contributes significantly to the knowledge base regarding the level and distribution of genetic diversity in the “Occidental” and “Oriental” eggplant gene pool, and sets the scene for a well-founded association mapping exercise to derive genotype-phenotype relationships.
